# Oral pretreatment with β-lactoglobulin derived peptide and CpG co-encapsulated in PLGA nanoparticles prior to sensitizations attenuates cow’s milk allergy development in mice

**DOI:** 10.3389/fimmu.2022.1053107

**Published:** 2023-01-06

**Authors:** Mengshan Liu, Suzan Thijssen, Wim E. Hennink, Johan Garssen, Cornelus F. van Nostrum, Linette E. M. Willemsen

**Affiliations:** ^1^ Division of Pharmaceutics, Utrecht Institute for Pharmaceutical Sciences, Utrecht University, Utrecht, Netherlands; ^2^ Division of Pharmacology, Utrecht Institute for Pharmaceutical Sciences, Utrecht University, Utrecht, Netherlands; ^3^ Department of Immunology, Nutricia Research B.V., Utrecht, Netherlands

**Keywords:** β-lactoglobulin, cow’s milk allergy, CpG oligodeoxynucleotides, individual ventilated cages, poly (lactic-co-glycolic acid) nanoparticles, toll-like-receptor 9, whey protein, oral pretreatment

## Abstract

Cow’s milk allergy is a common food allergy among infants. Improved hygiene conditions and loss of microbial diversity are associated with increased risk of allergy development. The intestinal immune system is essential for oral tolerance induction. In this respect, bacterial CpG DNA is known to drive Th1 and regulatory T-cell (Treg) development *via* Toll-Like-Receptor 9 (TLR-9) signaling, skewing away from the allergic Th2 phenotype. We aimed to induce allergen specific tolerance *via* oral delivery of poly (lactic-co-glycolic acid) nanoparticles (NP) co-encapsulated with a selected β-lactoglobulin derived peptide (BLG-Pep) and TLR-9 ligand CpG oligodeoxynucleotide (CpG). *In vivo*, 3-4-week-old female C3H/HeOuJ mice housed in individually ventilated cages received 6-consecutive-daily gavages of either PBS, whey, BLG-Pep/NP, CpG/NP, a mixture of BLG-Pep/NP plus CpG/NP or co-encapsulated BLG-Pep+CpG/NP, before 5-weekly oral sensitizations with whey plus cholera toxin (CT) or only CT (sham) and were challenged with whey 5 days after the last sensitization. The co-encapsulated BLG-Pep+CpG/NP pretreatment, but not BLG-Pep/NP, CpG/NP or the mixture of BLG-Pep/NP plus CpG/NP, prevented the whey-induced allergic skin reactivity and prevented rise in serum BLG-specific IgE compared to whey-sensitized mice. Importantly, co-encapsulated BLG-Pep+CpG/NP pretreatment reduced dendritic cell (DC) activation and lowered the frequencies of PD-L1+ DC in the mesenteric lymph nodes compared to whey-sensitized mice. By contrast, co-encapsulated BLG-Pep+CpG/NP pretreatment increased the frequency of splenic PD-L1+ DC compared to the BLG-Pep/NP plus CpG/NP recipients, in association with lower Th2 development and increased Treg/Th2 and Th1/Th2 ratios in the spleen. Oral administration of PLGA NP co-encapsulated with BLG-Pep and CpG prevented rise in serum BLG-specific IgE and symptom development while lowering splenic Th2 cell frequency in these mice which were kept under strict hygienic conditions.

## Introduction

Cow’s milk allergy (CMA) is one of the most common food allergies occurring early in life, with an overall incidence of 0.54% in Europe (1). Despite most infants can outgrow CMA, affected children need to avoid ingestion of cow’s milk proteins, which may restrict nutrient intake and decrease their quality of life. Early introduction of peanuts, between 4 and 11 months after birth, was found effective in prevention of peanut allergy development among high-risk infants (2) in the Learning Early About Peanut Allergy (LEAP) study. Similarly, early introduction of egg proteins between 4 and 6 months of age protected against the development of egg allergy (3). However, early introduction of cow’s milk protein was not found to reduce the risk on cow’s milk allergy among the general population in the Enquiring About Tolerance (EAT) observational study (4). Nevertheless, an earlier timing for cow’s milk protein exposure among a general population, in addition to breast feeding, within 14 days after birth (5) and during the first 3 months of life was found to be associated with a decreased risk of CMA (6).When breastfeeding is not possible, cow’s milk formula or hydrolyzed formula milk might be given to the atopic children at risk of CMA development as breastmilk substitute (7). However, neither cow’s milk formula nor hydrolyzed formula milk was proven effective in CMA prevention (7). Furthermore, early introduction of cow’s milk proteins (*i.e.* whole whey protein) might induce adverse reactions among infants that have been sensitized *via* environmental exposure *via* skin (8). Therefore, when breastfeeding is not possible for the high-risk infants, it is of importance to implement a safe and effective approach for CMA prevention. Previously 18-AA long synthetic peptides were identified within the β-lactoglobulin (BLG) sequence and shown to contribute to oral tolerance induction (9). In pre-clinical studies, BLG derived peptides showed reduced sensitizing capacity as compared to BLG protein (10), and were found capable of inducing oral tolerance to whole whey protein (9, 11). These peptides are too small to provoke allergic symptoms and therefore safe to provide early in life in children at risk to enrich formula milk. Our previous study showed a protective effect of BLG derived peptides encapsulated poly(lactic-*co*-glycolic acid) (PLGA) nanoparticles (NP) against CMA development when mice were housed in open cages (12). The gut-associated lymphoid tissue (GALT) is prone to induce peripheral immune tolerance to harmless food proteins (13) in the presence of the proper environmental stimuli as co-factors (14). Allergen loaded PLGA nanoparticles have been extensively exploited *via* subcutaneous (15) or oral route (16–18) for allergen immunotherapy in mice. In early life, microbial triggers are suggested to play a pivotal role in immune maturation and development of regulatory T-cells contributing to oral tolerance induction (19). Noteworthy, bacterial CpG DNA can activate the TLR-9 that is expressed in the endolysosomes of plasmacytoid DC and B-cells (20, 21) and drive regulatory immune maturation (22), skewing away from the allergic type 2 phenotype (23, 24). However, modern lifestyle and environmental changes, including improved hygiene conditions and use of antibiotics affect intestinal microbial diversity and commensal microbiota composition contributing to an immune imbalance and a rise in allergic diseases including food allergy (25, 26).

Housing mice in individual ventilated cages (IVC) simulates a strict hygienic environment with reduced exposure to bacterial ligands that are essential for immune function and/or contribute to immune maturation (27). We hypothesized that co-encapsulating TLR-9 ligand CpG together with a selected model synthetic peptide of 18-AA derived from BLG sequence (BLG-peptide, abbreviated as BLG-Pep) (12) in PLGA nanoparticles would improve the allergy preventive capacity of the BLG-Pep as CpG acts as a Th1 and Treg adjuvant (28–30). Noteworthy, this BLG-Pep was shown previously to be recognized by a cow’s milk specific human T-cell line *in vitro* and to reduce allergic skin reactivity to whole whey protein in mice when used in a relatively high dose (31) and at a lower dose when encapsulated in PLGA nanoparticles to optimize oral delivery (32). To further enhance the efficacy of this approach a strategy was developed to co-encapsulate BLG-Pep, to instruct allergen specificity, together with CpG, as an immunomodulatory adjuvant, into PLGA nanoparticles.

Previously, co-administration of antigen and class B CpG-oligonucleotides (CpG), as a Th1-skewing immune adjuvant, has been harnessed in food allergy (24) and allergic asthma immunotherapy (33) in mice. Current reported strategies for antigen and CpG co-delivery using PLGA carriers either incorporates cationic lipid dioleoyl-3-trimethylammonium propane (DOTAP) (34) or protamine (35). However, DOTAP functions as a Toll-like receptor 4 (TLR-4) agonist and interferes with the tolerance induction outcome. To preclude the influences from DOTAP and additional protein protamine, we have prepared well-defined CpG and BLG-Pep co-encapsulated PLGA nanoparticles.

In this study, we investigated the effect of orally administered nanoparticles co-encapsulated with CpG and BLG-Pep as a model peptide to prevent whey induced cow’s milk allergy in mice kept in IVC housing to mimic increased hygienic conditions.

## Materials and methods

### Peptide and CpG

A 18-AA-long sequential synthetic peptide that are derived from the chain B of bovine β-lactoglobulin, BLG-Pep (AASDISLLDAQSAPLRVY), previously indicated as Peptide 3 (12), was purchased from JPT Peptide Technologies (Berlin, Germany). Murine TLR-9 ligand CpG oligonucleotides 1826 (5’-tccatgacgttcctgacgtt-3’, further abbreviated as CpG) was purchased from *In vivo*Gen (San Diego, USA).

### Preparation and characterization of BLG-Pep and/or CpG encapsulated PLGA nanoparticles and release studies

Nanoparticles were prepared with poly (lactic-*co*-glycolic acid) (PLGA: lactide/glycolide molar ratio 50:50, 0.32 - 0.48 dL/g; PURASORB PDLG 5004A, Corbion, the Netherlands) using a double emulsion solvent evaporation method, as described in detail in the supplemental information. BLG-Pep (previously indicated as Peptide 3) were encapsulated in PLGA nanoparticles for the animal study as previously published (12, 32). The *in vitro* release method of BLG-Pep and CpG from the PLGA NP is described in detail in the supplemental method.

### Animal study

Sixty 3-4-week-old pathogen free female C3H/HeOuJ mice were ordered from Charles River Laboratories (Sulzfeld, Germany). The mice were randomly allocated into 7 treatment groups and housed in individually ventilated cages in the animal facility of Utrecht University. The mice were fed cow’s milk protein free control purified diet AIN-93G (contains soy protein and supplemented methionine to replace casein as protein source, Ssniff diet obtained *via* Bio-services, Uden, the Netherlands) *ad libitum* in the study. Animal care and use in this study follows the guidelines of the Animal Ethics committee and the Center Commission for Animal use (CCD) of Utrecht University, with approval numbers of AVD108002015262-2.

### Oral tolerance induction, sensitization and challenge

**Figure 1:**
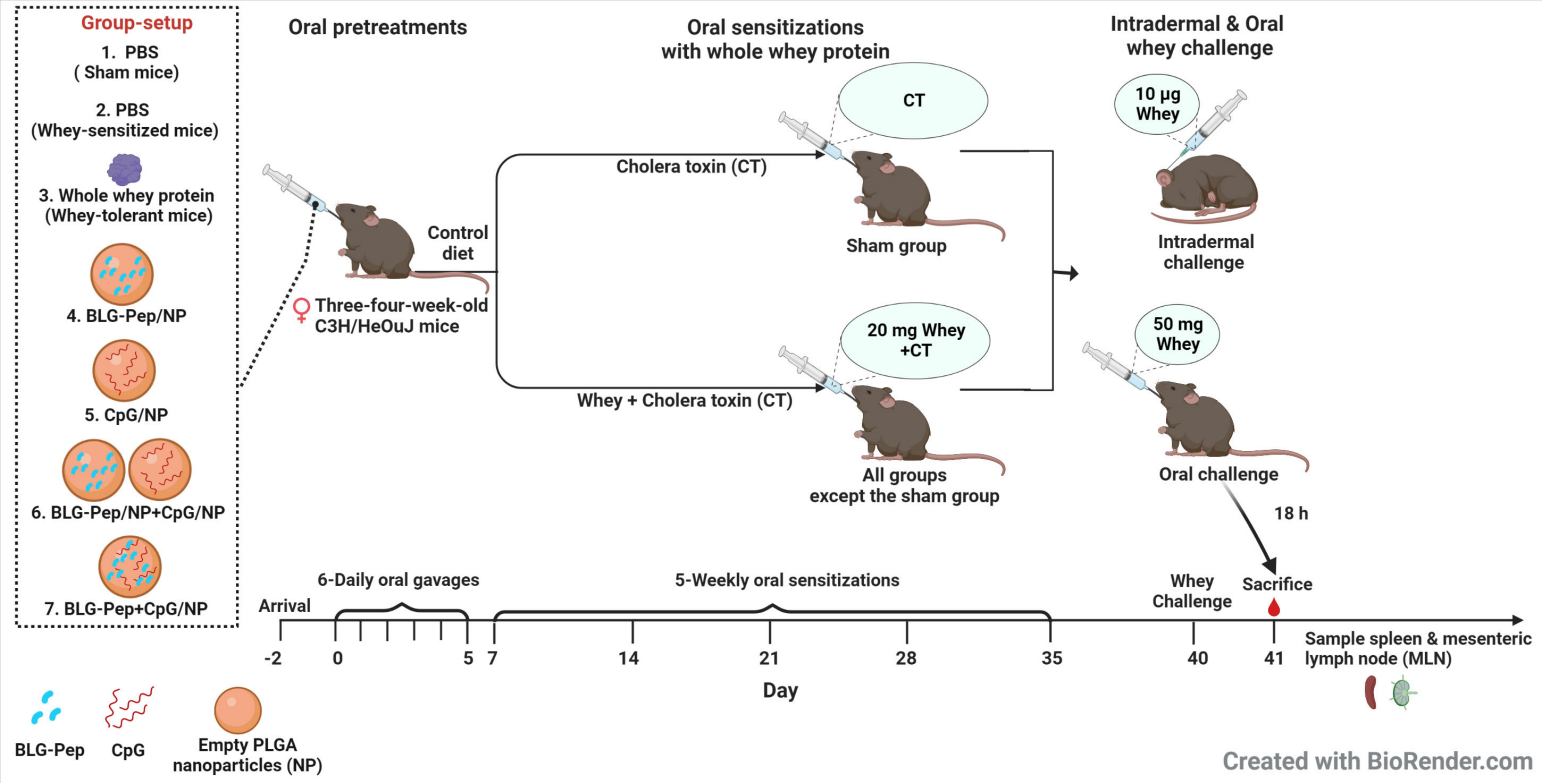
Experimental protocol for pre-treatment, sensitizations and challenge in murine model of cow’s milk allergy for the animal study. Three-four-week-old pathogen free female C3H/HeOuJ mice were given 6 daily oral pretreatments starting 2 days after arrival of the mice. Two days after the tolerance induction phase mice received 5-consecutive-weekly sensitizations with 20 mg whey plus 10 µg cholera toxin in 0.5 mL PBS for all groups but sham (only cholera toxin) from day 7 to day 35 to break whey tolerance. Five days after the last whey-sensitization, mice received intradermal challenge with 10 µg whole whey protein in the ears and at t=0 h and t=1 h the acute allergic skin response (ear swelling) was determined. Three hours after the intradermal whey challenge, the mice were orally challenged by means of gavage with 50 mg whey in 0.5 mL PBS. Eighteen hours after the oral whey challenge, mice were anesthetized with isoflurane and euthanized after blood sampling. Subsequently, spleen and mesenteric lymph nodes were collected immediately for further analysis. CT, Cholera toxin; NP, nanoparticles.

The CMA prevention murine model was developed and described by Schouten et al. (36) and Kostadinova et al. (37). As indicated in **Figure 1**, mice were randomly allocated into 7 groups and received 6-consecutive-daily oral pretreatments (**Table 1**) with the selected model BLG peptide (BLG-Pep) and/or CpG encapsulated in PLGA NP in a sterile biosafety cabinet starting 2 days after their arrival. Briefly, the mice received either PBS (Dulbecco’s phosphate-buffered saline, Sigma-Aldrich, Zwijdrecht, the Netherlands), 50 mg whey, BLG-Pep/NP (160 µg encapsulated BLG-Pep), CpG/NP (3 µg encapsulated CpG), a mixture of BLG-Pep/NP plus CpG/NP (160 µg BLG-Pep and 3 µg CpG in separate encapsulation form), or co-encapsulated BLG-Pep+CpG/NP (160 µg BLG-Pep and 3 µg CpG in co-encapsulation form) in 0.5 mL PBS *via* oral gavages. Two days after the tolerance induction phase mice received 5-consecutive-weekly sensitizations with 20 mg whey plus 10 µg cholera toxin (CT) in 0.5 mL PBS for all groups except sham (only CT) to break oral tolerance for whey from day 7 to day 35. Five days after the last whey-sensitization, mice received intradermal challenge with 10 µg whole whey protein in the ears (day 40) and at t=0 h and t=1 h the ear swelling response was measured (acute allergic skin response). Three hours after the intradermal whey challenge, the mice were orally challenged by means of gavage with 50 mg whey in 0.5 mL PBS. Eighteen hours after the oral whey challenge, mice were anesthetized with isoflurane and euthanized after blood sampling.

**Table 1:** Treatment groups of the animal study in the CMA prevention murine model.

Group	Pretreatment per mouse per dose	Sensitization	Challenge
Sham (n=4)	PBS	Cholera Toxin	Whey
Whey-sensitized (n=10)	PBS	Cholera Toxin plus Whey	Whey
Whey-tolerant (n=6)	Whey		Whey
BLG-Pep/NP (n=10)	23 mg BLG-Pep/NP (encapsulating 160 µg BLG-Pep plus 5 mg Empty NP)		Whey
CpG/NP (n=10)	5 mg CpG/NP (encapsulating 3 µg CpG plus 23 mg Empty NP)		Whey
BLG-Pep/NP+CpG/NP (n=10)	23 mg BLG-Pep/NP plus 5 mg CpG/NP (encapsulating 160 µg BLG-Pep and 3 µg CpG)		Whey
BLG-Pep+CpG/NP (n=10)	20 mg BLG-Pep+CpG/NP (encapsulating 160 µg BLG-Pep and 3 µg CpG)		Whey

### Assessment of acute allergic skin response

Five days after the last oral sensitization, the acute allergic skin response was recorded with the cage code concealed. Under anaesthesia by inhalation of isoflurane, the ear pinnae thickness of the mice was measured twice using a digital micrometer (Mitutoyo, Veenendaal, the Netherlands), before and 1 h after intradermal ear challenge with 10 µg whey protein in 20 µL PBS. Difference in ear pinnae thickness (ear swelling) was calculated by subtracting the basal ear pinnae thickness before challenge according to Schouten et al. (36). Meanwhile, body temperature and anaphylaxis symptoms score were recorded before and at 30 min and 1 h after intradermal challenge with whole whey protein according to Schouten et al. (36).

### Serum mucosal mast cell protease-1 and allergen specific immunoglobulins

mMCP-1 was measured in serum that was collected 18 h after the oral whey challenge, using the mouse mucosal mast cell protease-1 (MCPT-1) Elisa kit (eBioscience, ThermoFisher, Massachusetts, USA) according to the manufacturer’s protocol. Serum whey- and BLG-specific immunoglobulins were measured a previously reported method (31).

### Flow cytometry analysis of T cell subsets from spleen and DC from mesenteric lymph nodes and spleen

Eighteen hours after oral challenge of the mice with whole whey protein, isolation of cells from spleen (11) and mesenteric lymph nodes (MLN) (38) was performed as previously reported.

Approximate 8×10^5^ isolated MLN or spleen cells were resuspended in 135 µL PBS (Sigma-Aldrich) and cultured in 96-wells falcon plates. Cell viability was determined by staining with 100 µL fixable viability dye (FVD)-eFluor™780 (ThermoFisher) at 2000 times dilution in PBS at 4˚C. Nonspecific binding sites were blocked by incubation with 25 µL anti-mouse CD16/CD32 (Mouse BD Fc block; BD Pharmingen, San Jose, USA) at 100 times dilution in 2% Fetal Bovine Serum (FBS)-1% Bovine Serum Albumin (BSA, Sigma-Aldrich)-PBS buffer for 5 min at 4˚C.

For extracellular staining of Th1/Th2 and Treg (Antibodies concentrations are shown in the **Supplemental Tables 1, 2**), splenocytes were incubated with CD4-BV510 (BioLegend, San Diego, USA), T1/ST2-FITC (MD Biosciences, Oakdale, USA), CXCR3-PE (ThermoFisher) and CD25-PerCP/eFluor710 (ThermoFisher) in 1% BSA-PBS buffer overnight at 4˚C. For intracellular Treg staining, cells were first fixed and permeabilized with FoxP3/Transcription Factor Staining Buffer Set (ThermoFisher) and then stained with FoxP3-FITC (ThermoFisher).

For extracellular staining of DC (antibodies concentrations are shown in the **Supplemental Table 3**), MLN and spleen cell suspension were incubated with MHCII-PE (ThermoFisher), CD11c-FITC (ThermoFisher), CD11b-PE/Cy7 (ThermoFisher), CD80-BV421 (BioLegend), CD86-BV510 (BioLegend) and programmed cell death ligand 1 (PD-L1)-PerCP/Cy5.5 (ThermoFisher) 1% BSA-PBS buffer overnight at 4˚C. Cells were analyzed with BD FACSCantoII flow cytometer (Becton Dickinson, Franklin Lakes, USA). Data analysis was conducted with FlowLogic software (Inivai Technologies, Mentone, Australia).

### *Ex vivo* allergen re-stimulated cytokine release by splenocytes

Splenocytes (6×10^5^ cells) were stimulated with either 200 μL 500 µg/mL whey protein (DMV International, Veghel, the Netherlands), 290 µg/mL β-Lactoglobulin B variant (from bovine milk, Sigma-Aldrich), or only blank RPMI 1640 medium supplemented with 10% FBS, 100 U/mL penicillin and 100 μg/mL streptomycin as control and cultured in a round-bottom culture plate at 37°C, 5% CO_2_. After 5-days, supernatants were collected and determined using a Procartaplex kit for mouse IL-13, IL-5, IL-10 and TNF-α and mouse IFN-γ, IL-17A and TGF-β ELISA kits (both from ThermoFisher) according to the manufacturer’s protocols and using a GloMax^®^ Discover plate reader (Promega, Wisconsin, USA).

### Statistical analysis

The obtained *in vivo* data were analyzed with GraphPad Prism 9.0.0 software (GraphPad Software, San Diego, USA). Normal distribution of the data was assessed by the Shapiro-Wilk test and Kolmogorov-Smirnov test. If required data were log transformed to obtain normal distribution or alternatively a non-parametric test was used. One-way ANOVA followed by Bonferroni’s *post hoc* test, or the non-parametric Kruskal-Wallis test followed by Dunn’s *post hoc* test was applied for selected pairs (as indicated in the figure legends). In the *post hoc* analyses for selected pairs the whey-sensitized group was compared to all the other groups and the BLG-Pep+CpG/NP group was compared to all groups except the sham group (non-sensitized group).

## Results

### Characteristics of PLGA nanoparticles

Empty and encapsulated PLGA nanoparticles had average hydrodynamic particle sizes in the range of 240-290 nm. As a parameter that described the size range of nanocarriers (39), low polydispersity indices (PDI) below 0.14 (**Table 2**) of these nanoparticles demonstrated their narrow size distributions. The particles had close to neutral zeta-potentials (around -1 mV), as a key parameter reflects the electrostatic interactions and stability of the nanoparticles dispersions (40), in 10 mM HEPES buffer (pH7.4). BLG-Pep was encapsulated without CpG (in BLG-Pep/NP) or with CpG (in BLG-Pep+CpG/NP) with encapsulation efficiencies (EE) between 41-65% and loading capacities (LC) between 0.7-0.8%. CpG was encapsulated in CpG/NP (*i.e.*, without peptide) and in BLG-Pep+CpG/NP with EEs between 38-47% and LCs between 0.02-0.06%. Quantification of the encapsulated BLG-Pep and CpG cargos in the PLGA nanoparticles enabled further *in vitro* release study and dosing for the pretreatments in the animal study.

**Table 2:** Characteristics of BLG-Pep and/or CpG encapsulated PLGA nanoparticles.

Formulation (Number of combined batches)	Hydrodynamic particle size (nm) (Measurement in triplicate)	Poly dispersity Index (PDI)	Zeta-potential^1^ (mV)	CpG		BLG-Pep	
				EE^2^ (%)	LC^3^ (%)	EE (%)	LC (%)
Empty NP (n=6)	250 ± 15	0.09 ± 0.07	-1.2 ± 0.2	–	-	–	–
BLG-Pep/NP (n=3)	264 ± 3	0.08 ± 0.02	-1.0 ± 0.3	–	-	65	0.7
CpG/NP (n=2)	245 ± 2	0.06 ± 0.04	-1.2 ± 0.4	47	0.06	–	–
BLG-Pep+CpG/NP (n=2)	242 ± 3	0.08 ± 0.03	-0.8 ± 0.6	38	0.02	41	0.8

^1^Zeta-potential: Zeta-potential of the PLGA nanoparticles were characterized in 10 mM HEPEs buffer at pH7.4.

^2^EE: Encapsulation Efficiency= $\;\frac{\text{Amount of encapsulated BLG}-\text{Pep or CpG}}{\text{Amount of feed BLG}-\text{Pep or CpG for fomulation preparation}}\times 100\;\left(\%\right)$

^3^LC: Loading Capacity= $\;\frac{\text{Amount of encapsulated BLG}-\text{Pep or CpG }}{\text{Weight of nanoparticles}}\times 100\;\left(\%\right)$ .

The *in vitro* release profiles of BLG-Pep/NP were similar to our previous study (12), except for a burst release of 18% BLG-Pep from the BLG-Pep/NP and no burst release of the peptide cargo from the BLG-Pep+CpG/NP used in the animal study (**Supplemental Figure 1A**). Thereafter, a slow-release phase and accelerated release phase of peptide cargo were observed during 49-58 days.

For the release of CpG, a burst release of 20% CpG from CpG/NP and 10% CpG from BLG-Pep+CpG/NP were observed, followed by a sustained release of CpG from day 7 onwards (**Supplemental Figure 1B**).

### Co-encapsulated BLG-Pep+CpG/NP prevented the acute allergic skin response and serum BLG-specific IgE levels

**Figure 2:**
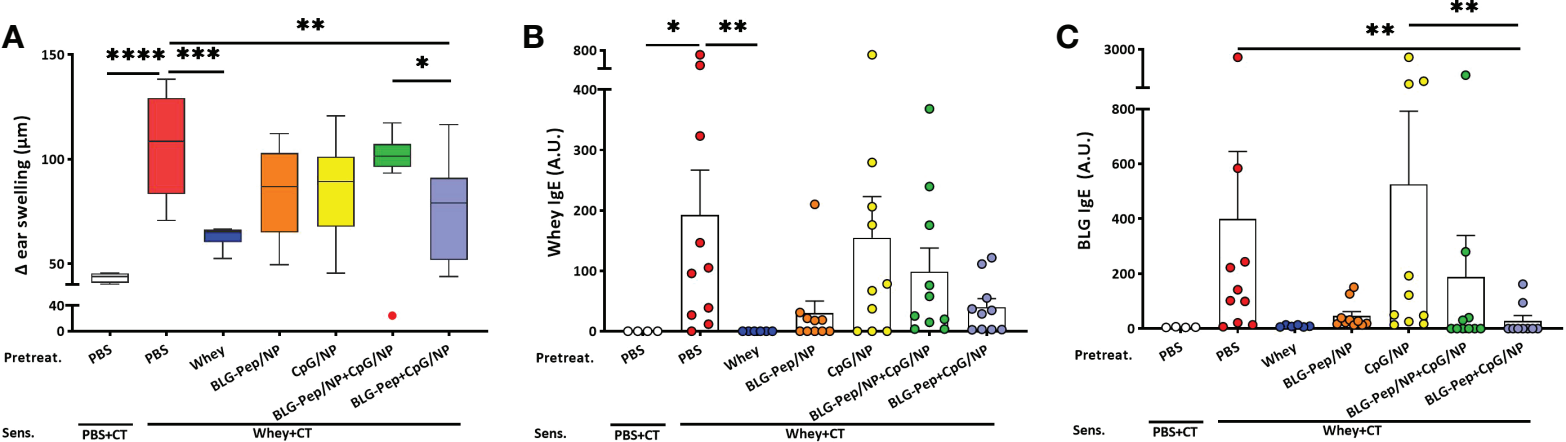
Acute allergic skin response and BLG- and whey-specific serum immunoglobulin E. Five days after last sensitization, mice were intradermally challenged in the ear pinnae with 10 µg whey followed by oral challenge. The acute allergic skin response was measured 60 min afterwards in the *in vivo* study **(A)**. BLG- **(B)** and whey-specific **(C)** IgE levels are measured in serum, which were collected 18 h after last oral challenge with whey in mice from all groups of the animal study. Data are presented as box-and-whisker Tukey plots for **(A)**, and mean ± SEM for **(B, C)** n=9-10 per group except for the sham group, n=4 and whey-tolerant group, n=6. **(A–C)** are presented with Y axis formatted in two segments to properly show the relevant part for the window of effect **(A)** or to be able to appreciate the full data set **(B, C)**. **(A)** is analyzed by one-way ANOVA, followed by Bonferroni’s *post hoc* test for selected pairs. The outlier (in red) in the BLG-Pep/NP+CpG/NP group is excluded in **(A)** from statistics; **(B, C)** are analyzed with the Kruskal-Wallis non-parametric test, followed by Dunn’s *post hoc* test for selected pairs; *p<0.05, **p<0.01, ***p<0.001, ****p<0.0001; BLG, β-lactoglobulin; CT, Cholera toxin.

The animal study was performed in IVC housing as indicated in **Table 1** and **Figure 1**. Whey-sensitized (sham-pretreated mice) mice had significantly increased ear thickness one hour after intradermal whey challenge, as compared to the sham (PBS-pretreated mice) and whey-tolerant group (whey-pretreated mice), indicating successful establishment of a prophylatic CMA murine model in this study (**Figure 2A** and **Supplemental Figure 2A**). Pretreatments by nanoparticles encapsulated with only BLG-Pep (BLG-Pep/NP) did not prevent the acute allergic skin response (**Figure 2A**). In the animal study, only co-encapsulated BLG-Pep+CpG/NP, but not pretreatments with, PBS (sham group), BLG-Pep/NP, CpG/NP or BLG-Pep/NP+CpG/NP, effectively prevented the whey induced acute allergic skin response as compared to the whey-sensitized (sham-pretreated) mice. Moreover, co-encapsulated BLG-Pep+CpG/NP prevented the whey induced acute allergic skin response as compared to separately delivered BLG-Pep/NP+CpG/NP in mice (**Figure 2A** and **Supplemental Figure 2A**). No statistic difference was observed in body temperature or anaphylactic shock score of the mice during 1 h after intradermal whey challenge (**Supplemental Figure 3**). Despite lack of statistical significance, mMCP-1 measured in the serum showed a similar pattern as the acute allergic skin response result (**Supplemental Figure 2B**).

Consistent with the prevented allergic skin reactivity, mean BLG-specific IgE serum levels 18 h after oral whey challenge were significantly lower in the BLG-Pep+CpG/NP recipients compared to both whey-sensitized (sham-pretreated mice) and CpG/NP groups (**Figure 2B**). A similar overall pattern was found for whey-specific IgE, albeit this did not reach statistical significance (**Figure 2C**). In the whey-tolerant group (whey-pretreated mice), BLG- and whey-specific IgG1 and IgG2a remained low (**Supplemental Figure 4A–D**), but the other pretreatments did not reduce these immunoglobulins levels.

### In mesenteric lymph nodes co-encapsulated BLG-Pep+CpG/NP decreased percentages of CD80/CD86+CD11b+ DC

**Figure 3:**
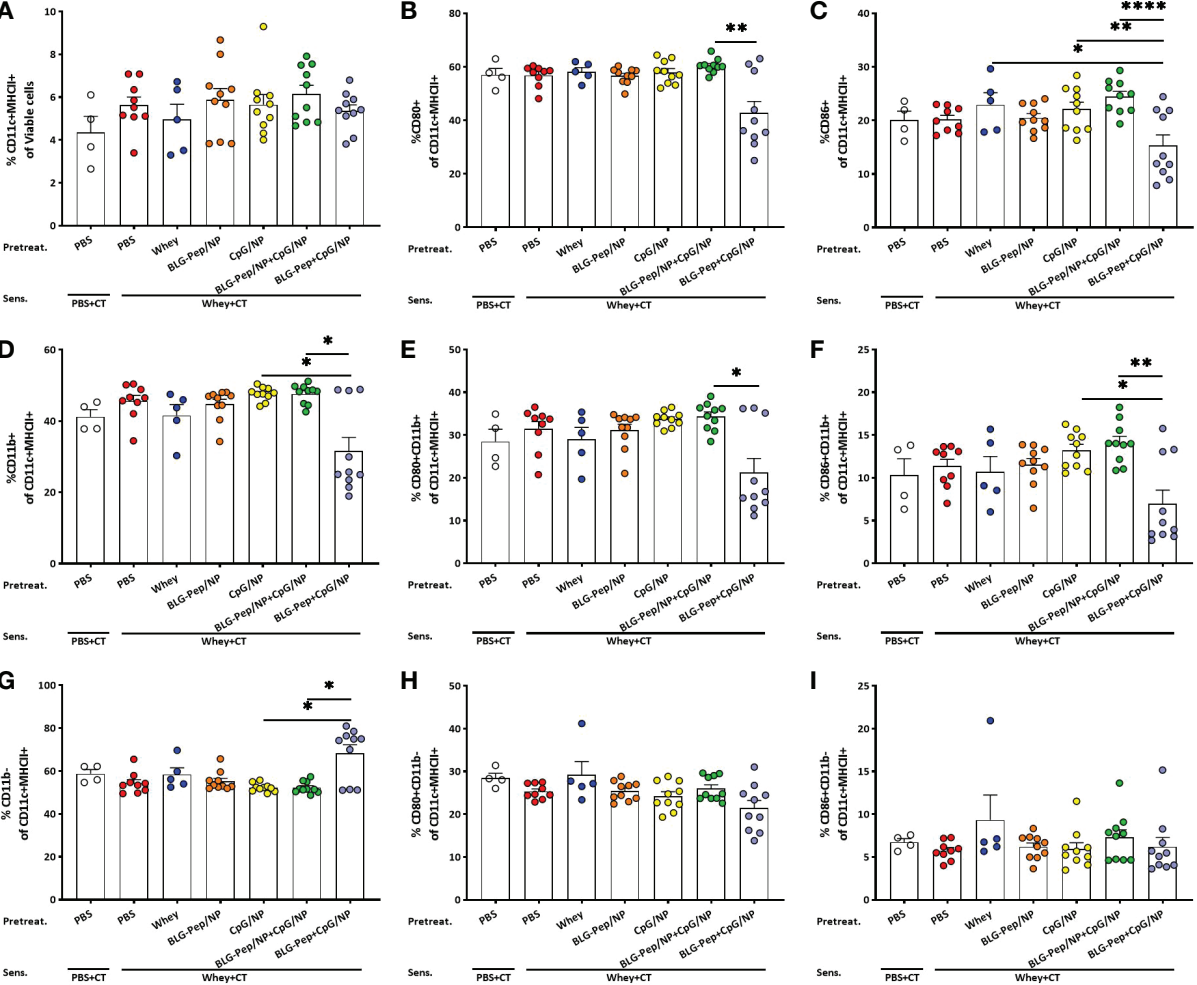
Surface activation markers expression on dendritic cells in mesenteric lymph nodes (MLN). Eighteen hours after oral whey-sensitization, MLN cells were isolated and analyzed using flow cytometry to determine the percentage of CD11c+MHCII+ DC **(A)** and CD11b+ **(D)** and CD11b- **(G)** DC subsets. Surface costimulatory molecules expression of CD80+ **(B, E, H)** and CD86+ **(C, F, I)** on CD11c+MHCII+ DC, CD11b+ and CD11b- DC subsets were determined respectively. Data are presented as mean ± SEM for n=9-10 per group except for the sham group, n=4 and whey-tolerant group, n=5. **(A)** is analyzed with one-way ANOVA for selected pairs after log transformation, followed by Bonferroni’s *post hoc* test; **(B)** and **(D–I)** are analyzed with the Kruskal-Wallis non-parametric test, followed by Dunn’s *post hoc* test for selected pairs; **(C)** is analyzed with one-way ANOVA for selected pairs after log transformation, followed by Bonferroni’s *post hoc* test; *p<0.05, **p<0.01; CT, Cholera toxin. ****p<0.0001.

No differences in the mean percentages of CD11c+MHCII+ DC in MLN were found among all pretreatment groups (**Figure 3A**). The co-encapsulated BLG-Pep+CpG/NP recipients showed a lower percentage CD11b+ DC and higher percentage of CD11b- DC subsets compared to both the CpG/NP group and BLG-Pep/NP+CpG/NP group (**Figures 3D, G**). The co-encapsulated BLG-Pep+CpG/NP pretreatment reduced the expression of costimulatory molecules CD80 and CD86 on DC and CD11b+ DC compared to BLG-Pep/NP+CpG/NP pretreatment, but not on CD11b- DC in MLN (**Figures 3B, C, E, F, H** **, I**). The gating strategy of the flow cytometry analysis for DC from MLN is shown in **Supplemental Figure 8**.

### Co-encapsulated BLG-Pep+CpG/NP reduced percentages of PD-L1+ DC subsets in mesenteric lymph nodes but increased PD-L1+CD11b+ DC frequency in spleen

**Figure 4:**
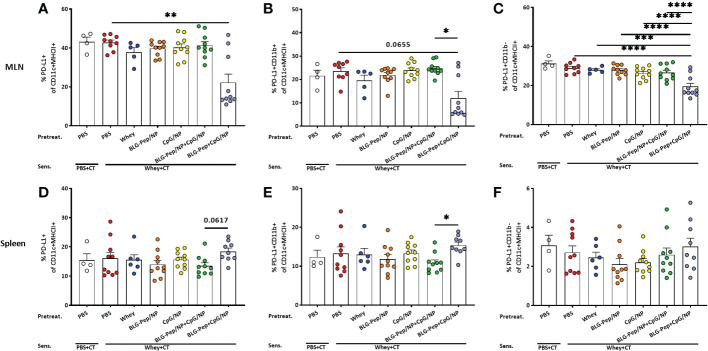
Expression of PD-L1 on dendritic cells from mesenteric lymph nodes (MLN) and spleen. Eighteen hours after the last oral challenge with whey, cells isolated from MLN and spleen were analyzed by flow cytometry for the frequencies of PD-L1+ on CD11c+MHCII+ DC **(A, D)**, PD-L1+CD11b+ **(B, E)** and PD-L1+CD11b- **(C, F)** DC subsets in MLN and spleen respectively. Data are presented as mean ± SEM for n=8-10 per group except for the sham group, n=4 and whey-tolerant group, n=6. **(A, B)** and **(D–F)** are analyzed with the Kruskal-Wallis non-parametric test, followed by Dunn’s *post hoc* test for selected pairs; **(C)** is analyzed with one-way ANOVA for selected pairs after log transformation, followed by Bonferroni’s *post hoc* test; *p<0.05, **p<0.01; CT, Cholera toxin. ***p<0.001, ****p<0.0001.

In MLN, co-encapsulated BLG-Pep+CpG/NP, but not the other pretreatments, significantly decreased surface expression of PD-L1+ on CD11c+MHCII+ DC (**Figure 4A**) as compared to the whey-sensitized group in CD11b- DC (**Figure 4C**), and only showed a decreasing tendency in CD11b+ DC subset (*p*=0.0655, **Figure 4B**). In spleen, however, co-encapsulated BLG-Pep+CpG/NP pretreatment upregulated expression of PD-L1 on CD11b+ DC subset (**Figure 4E**) and tended to enhance PD-L1 expression on CD11c+MHCII+ DC (*p*=0.0617, **Figure 4D**), but not on its CD11b- DC subset (**Figure 4F**), as compared to the BLG-Pep/NP plus CpG/NP group.

### In splenocytes co-encapsulated BLG-Pep+CpG/NP lowered the percentage of Th2 cell while increasing ratios Treg/Th2 and Th1/Th2

**Figure 5:**
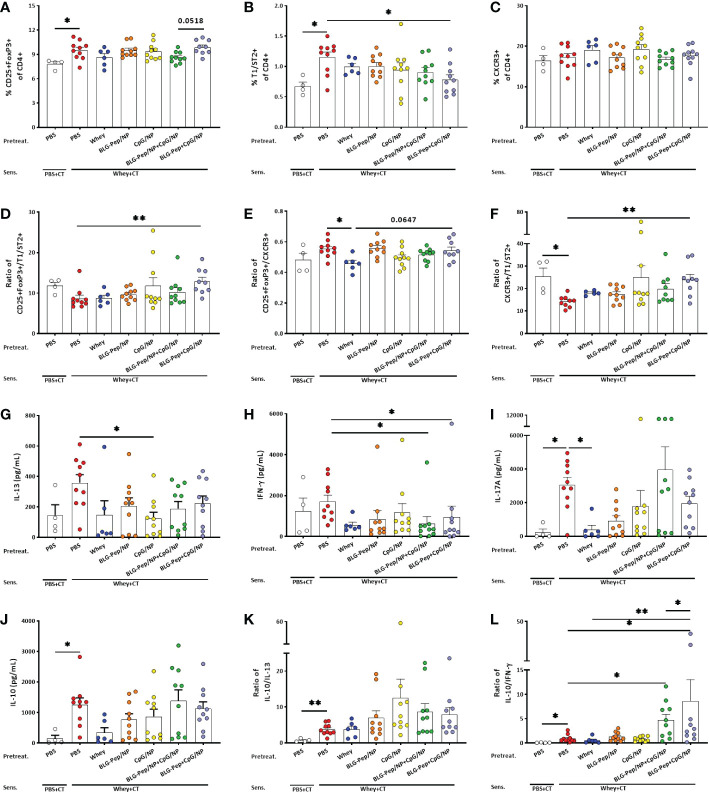
Splenic T-cell subsets and cytokines production of splenocytes upon *ex vivo* stimulation with whey for 5 days. Eighteen hours after the last oral challenge with whey, splenocytes were isolated and analyzed by flow cytometry for the percentages of T-cell subsets including Treg **(A)**, Th2 **(B)**, Th1 **(C)**, ratio of Treg/Th2 **(D)**, ratio of Treg/Th1 **(E)** and ratio of Th1/Th2 **(F)** respectively. Splenocytes were restimulated with whole whey protein for 5 days, Th2- **(G)**, Th1- **(H)**, Th17- **(I)**, and Treg- **(J)** associated cytokines were measured in supernatants. Ratios of Treg-/Th2- associated **(K)** and Treg-/Th1-associated **(L)** cytokines were calculated. **(F, I, K, L)** are presented with Y axis formatted in two segments to be able to appreciate the full data set. Data are presented as mean ± SEM for n=9-10 per group except for the sham group, n=4 and whey-tolerant group, n=6. **(A–C)** and **(E)** are analyzed with one-way ANOVA for selected pairs, followed by Bonferroni’s *post hoc* test; **(D)** and **(F–K)** are analyzed with the Kruskal-Wallis non-parametric test, followed by Dunn’s *post hoc* test for selected pairs; **(L)** is analyzed with one-way ANOVA for selected pairs after log transformation, followed by Bonferroni’s *post hoc* test; *p<0.05, **p<0.01; CT, Cholera toxin.

The co-encapsulated BLG-Pep+CpG/NP pretreatment resulted in an increasing trend in CD25+FoxP3+ Treg percentage as compared to the BLG-Pep/NP+CpG/NP pretreated mice (*p*=0.0518, **Figure 5A**).The co-encapsulated BLG-Pep+CpG/NP pretreatment reduced the percentage of T1/ST2+ Th2 cells (**Figure 5B**) compared to the whey-sensitized group, but no difference was found on the frequencies of CXCR3+ Th1 (**Figure 5C**) and CD196+ Th17 subsets (Data not shown for Th17) in all pretreatment groups. Co-encapsulated BLG-Pep+CpG/NP increased ratios of Treg/Th2 and Th1/Th2 as compared to the whey-sensitized mice (**Figures 5D, F**), and tended to increase the Treg/Th1 ratio in BLG-Pep+CpG/NP recipients as compared to whey-tolerant mice (*p*=0.0647, **Figure 5E**). The gating strategy of the flow cytometry analysis for Th1, Th2 and Treg subsets from splenocytes is shown in **Supplemental Figure 9**.

### Co-encapsulated BLG-Pep+CpG/NP decreased whey-restimulated IFN-γ and enhanced IL-10/IFN-γ ratio in splenocyte supernatants

CpG/NP pretreatment reduced whey-restimulated splenocytes release of IL-13 (**Figure 5G**), while BLG-Pep/NP+CpG/NP and co-encapsulated BLG-Pep+CpG/NP pretreatments lowered whey-restimulated splenocytes release of IFN-γ compared with the whey-sensitized mice (**Figure 5H**). Whey-sensitized mice showed higher whey-stimulated IL-17A and IL-10 production by splenocytes compared to sham mice (**Figures 5I, J**), which was prevented in the whey-tolerant group for IL-17A secretion (**Figure 5I**). Despite no statistical differences were found in IL-10 concentrations or in the ratio of IL-10/IL-13 (**Figure 5K**), BLG-Pep/NP+CpG/NP and co-encapsulated BLG-Pep+CpG/NP pretreatments significantly increased the ratio of IL-10/IFN-γ compared to whey-sensitized mice (**Figure 5L**). BLG-Pep+CpG/NP pretreatment also significantly increased the ratio of IL-10/IFN-γ compared to the BLG-Pep/NP+CpG/NP group and whey-tolerant group (**Figure 5L**). **Supplemental Figure 5–7** shown medium versus whey-stimulated cytokine responses and additional whey or BLG induced re-stimulation responses.

## Discussion

We hypothesized that CpG as an adjuvant can enforce the allergy preventive effect of a model selected BLG peptide co-encapsulated in PLGA (BLG-Pep/NP) under strict hygienic conditions. Previously, this selected BLG peptide (BLG-Pep) was shown to be recognized by a cow’s milk specific human T-cell line *in vitro* and to inhibit acute allergic skin response to whey protein in mice pretreated with a relatively high dose (31) and at a lower dose when encapsulated in PLGA nanoparticles to optimize oral delivery (32). In this study, 3-4-week-old female mice were chosen to investigate the oral tolerance induction in early life using pretreatment of BLG-peptide and CpG co-encapsulated PLGA NP before oral sensitization with whole whey protein. In the 3-4-week-old mice, microfold (M) cells have already reached adult levels, since this is achieved before 2-3 week of age. This will allow access of the PLGA nanoparticles to the Peyer’s patch and gut draining mesenteric lymph nodes where nanoparticle uptake and peptide presentation by the DC can instruct T-cell development (41, 42). Recruitment of T-cells to the neonatal mucosa (43, 44) and microbiome maturation (45) are pivotal for induction of Treg and thus establishment of oral tolerance (46). To further enhance the allergy preventive effect, a strategy was developed to co-encapsulate BLG peptide (to instruct allergen specificity) together with CpG (as an immunomodulatory adjuvant) into PLGA nanoparticles.

BLG-Pep and CpG were separately dissolved in the water phase (PBS), and both were emulsified with PLGA solubilized in oil phase (dichloromethane) to form two water-in-oil emulsions. This was followed by combining these two water-in-oil emulsions and next *via* preparing the water-in-oil-in-water emulsion (47) in order to co-encapsulate BLG-Pep and CpG into the PLGA nanoparticles. BLG-Pep and CpG were co-encapsulated in ~250 nm size PLGA nanoparticles with encapsulation efficiencies for both compounds of about 40%. *In vitro*, the BLG-Pep+CpG/NP showed a burst release of about 20% of the CpG, and no burst release of BLG-Pep from the NP matrix, which might be attributed to the insufficient washing and/or diffusion from the microporous channels that were resulted from the freeze drying process (48, 49). The sustained release of BLG-Pep+CpG/NP from PLGA NP demonstrates that a suitable formulation for *in vivo* evaluation was developed. Similar to many other drugs, there is a therapeutic dose window for CpG as an adjuvant, and toxicity was shown in mice that received a repetitive daily injection of CpG in a dose above 2.4 mg/kg (50). In this study, a relative low dose of 3 μg encapsulated CpG per daily oral gavage was given to the mice for a period of 6 days, which is a safe dosing scheme in terms of toxicity as reviewed (51). PLGA is biodegradable and biocompatible polymer as approved by the US Food and Drug Administration (FDA) for vaccine and drug delivery (52, 53). PLGA nanopaticles provide protection for the encapsulated BLG-peptide and CpG cargos (54) and facilitate cellular uptake by antigen-presenting cells (54).

**Figure 6:**
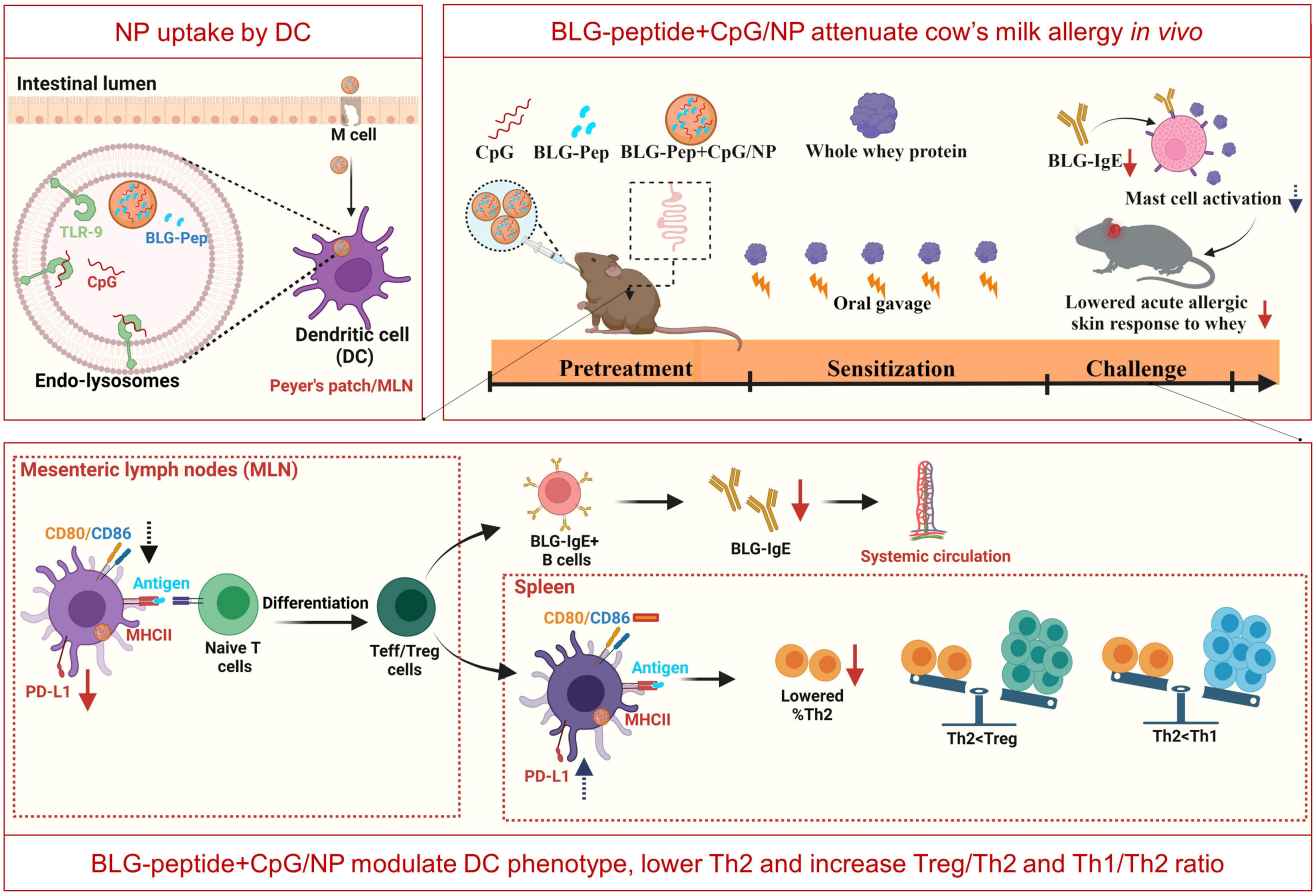
Graphical Abstract (Created with BioRender.com).

As shown by previous studies, intestinal uptake and lymphatic transport of oral delivered nanoparticles are dependent on surface charge, particle size and dose of the administered nanoparticles (55–57). The similar size and surface charge of the different PLGA NP suggest similar mucus penetration and cellular uptake of these nanoparticles after oral administration. Despite that, only the co-encapsulation gave the allergy preventive effect (*i.e*., prevented acute allergic skin reactivity and rise in BLG-specific IgE) even compared to the cocktail of BLG-Pep/NP plus CpG/NP. Antigen-encapsulated PLGA nanoparticles can be internalized by DC into phagosomes *via* endocytosis (58), followed by endosomal degradation for the MHCII pathway (59). On the other hand, TLR-9 and MHCII are also present in the endolysosomes of plasmacytoid DC and B-cells (21, 30). Thus, simultaneous internalization and intracellular release of CpG and BLG-Pep from BLG-Pep+CpG/NP in the endolysosomes of DC may facilitate synergistic activation of TLR-9 and antigen-presentation *via* MHCII pathway in the same DC (see graphical abstract **Figure 6**).

Oral pretreatment with a daily dosage of both antigen and adjuvant (160 μg BLG-Pep plus 3 μg CpG) for a short period (6 days) prevented skin reactivity and rise in serum BLG-specific IgE. Srivastava et al. (24) reported oral therapy using whole peanut extract encapsulated PLGA nanoparticles with surface conjugation of CpG, inhibited peanut allergic symptoms and facilitated peanut allergy outgrowth (24). Our study differs from this study substantially in that we investigated the cow’s milk allergy prevention effect using BLG-Pep (18-AA) and CpG co-encapsulated PLGA nanoparticles in a cow’s milk allergy murine model kept in IVC housing. In this study, BLG-Pep+CpG/NP pretreatment significantly prevented rise in BLG-specific IgE levels, but did not lower whey- or BLG-specific IgG1 and IgG2a levels. Despite the role of allergen-specific IgG1 in allergic diseases remains elusive (60), it is likely that full inhibition of allergen-specific IgE and IgG for both BLG and whey requires additional whey epitopes. Here we used only one small BLG model peptide of 18-AA while BLG protein itself consist of 162-AA (18.4 kDa) and multiple epitopes (61). Moreover, whole whey protein consists not only of BLG as allergen but also other allergenic proteins such as alpha-lactoglobulin (61). Thus, in future studies, an additional BLG-peptide from another region in the BLG protein sequence and/or peptides of other whey-derived allergens may be supplemented to the current regime and co-encapsulated with CpG to generate full tolerance for whole whey protein.

DC as antigen presenting cells, capture and recognize microorganisms *via* pathogen-associated-molecular-patterns (PAMPs) by Toll-Like Receptors (TLRs) (62), mediating tolerance (63) or activation of T-cell responses (64) to various antigens. Co-encapsulated BLG-Pep+CpG/NP recipients showed a lower frequency of the Th2/Th17 immune polarizing CD11b+ DC subset in the MLN (65), and a reduced expression of costimulatory molecules CD80/CD86 on CD11c+MHCII+ DC compared to the BLG-Pep/NP plus CpG/NP group, suggesting a more tolerogenic DC phenotype and consequent constraint of Th2 immunity (66). Programmed cell death ligand 1 (PD-L1) is expressed on non-lymphoid and lymphoid cells, including resting B-cells, T-cells, macrophages and DC, and regulates immune responses in secondary lymphoid and target organs (67). In particular, expression of PD-L1 on DC was reported essential for inducing IL-10 secreting T-cells *in vitro* (68), and required for generation of adaptive Treg subsets both *in vitro* and *in vivo* (69). Despite the diminished frequency of PD-L1+ DC in the MLN of the BLG-Pep+CpG/NP recipients, the simultaneous decreased surface expression of co-stimulatory molecules CD80/CD86 was previously shown to enforce a tolerogenic phenotype with a reduced maturation status (70).

Given the short lifespan of DC for less than 9 days (71), we speculate that the general DC phenotype may have undergone epigenetic changes and form immune memory responses *via* a process known as trained immunity resulting in a more tolerogenic DC phenotype (72, 73). Even though BLG-Pep+CpG/NP pretreatment reduced PD-L1 expression in the MLN compared to the whey-sensitized mice and the BLG-Pep/NP plus CpG/NP recipients, it enhanced the PD-L1 expression on splenic CD11b+ DC as compared to BLG-Pep/NP plus CpG/NP recipients. The latter was associated with an increase in the percentage of CD25+FoxP3+ Treg cells compared to the BLG-Pep/NP plus CpG/NP group. We hypothesize BLG-Pep+CpG/NP pretreatment to have instructed development of Treg that reside in the spleen, upon reactivation these cells may modify the phenotype of local DC resulting in enhanced PD-L1 expression actively contributing to systemic Treg function and a tolerogenic micromilieu (74–77). Alternatively, the BLG-Pep+CpG/NP pretreatment may have resulted in upregulation of PD-L1 on intestinal DC upon whey challenge, which than may have migrated *via* the MLN to the spleen for antigen presentation to naive CD4+ T-cells (78, 79), inducing development of regulatory T-cell and memory T-cells (74–77).

Indeed, a high dose CpG (50 μg) was reported to enhance expression of indoleamine 2,3-dioxygenase (IDO) in the plasmacytoid DC (80) after ligation to the Toll-like Receptor-9 in the endolysosomes, and hence activate a suppressor phenotype of Treg cells for tolerance induction (81). Therefore, we hypothesize the PLGA nanoparticles co-encapsulating only 3 µg CpG and 160 µg BLG-Pep might generate a tolerogenic micromilieu preventing allergy development for whole whey protein *via* supporting regulatory responses. The provided dose of CpG was relatively low and already effective. The dose-dependency of the BLG-Pep and CpG co-encapsulated PLGA NP for allergy prevention and antigen-specific tolerance induction *via* oral delivery and possible uptake by plasmacytoid DC warrants further investigation.

As reported, CD11b+ DCs initiate and maintain Th2 immunity towards allergens (82). However, BLG-Pep+CpG/NP pretreatment upregulated PD-L1 expression on these conventional CD11b+ splenic DC compared to BLG-Pep/NP plus CpG/NP recipients, which might also have promoted Treg development and reduced Th2 immunity upon re-exposure to whey allergens during sensitization and challenge stages (83, 84). Indeed, BLG-Pep+CpG/NP pretreatment induced a significant diminished frequency of T1/ST2+ Th2 cells, and higher splenic Treg/Th2, Th1/Th2 and IL-10/IFN-γ ratios compared to the whey-sensitized mice, which may favor a tolerogenic outcome (see graphical abstract **Figure 6**). The observed constraint of Th2 cells development might be attributed to the immunomodulatory effect from the co-encapsulated Type B CpG. As reported previously (85), systemic administered CpG reduces Th2 associated cytokines production without affecting Th1 cytokines in a murine asthma model. However, in the current study despite the decreased Th2 frequency in the CpG and peptide co-encapsulated group, upon BLG restimulation of the splenocytes the cytokine release was not silenced which was the case in the whey-tolerant group (**Supplemental Figure 7**). Hence, pretreatment with a single 18-AA BLG peptide may not be sufficient in overruling the immunogenic properties of the full immunogenic T-cell epitope repertoire of the BLG fraction in whey protein.

Collectively, these observations provide evidence that BLG-Pep+CpG/NP pretreatment changes the DC phenotype, reduces Th2 development and drives towards a Th1 and Treg microenvironment, which might contribute to reducing allergen-specific CMA development.

## Data availability statement

The original contributions presented in the study are included in the article/**Supplementary Material**. Further inquiries can be directed to the corresponding author.

## Ethics statement

The animal study was reviewed and approved by Animal Ethics committee of Utrecht University and the Center Commission for Animal use (CCD).

## Author contributions

LW, CN, and ML designed the experiments; ML and ST performed the experimental procedures. ML performed data collection and analyses and drafted the manuscript. LW, CN, WH, and JG contributed to data interpretation and critically revised the manuscript. All authors contributed to the article and approved the submitted version.
