# Supplementary figures and images for: Oral pretreatment with β-lactoglobulin derived peptide and CpG co-encapsulated in PLGA nanoparticles prior to sensitizations attenuates cow’s milk allergy development in mice
Source: Front Immunol. 2023 Jan 6;13:1053107. doi: 10.3389/fimmu.2022.1053107 (PMC9872660; doi:10.3389/fimmu.2022.1053107)

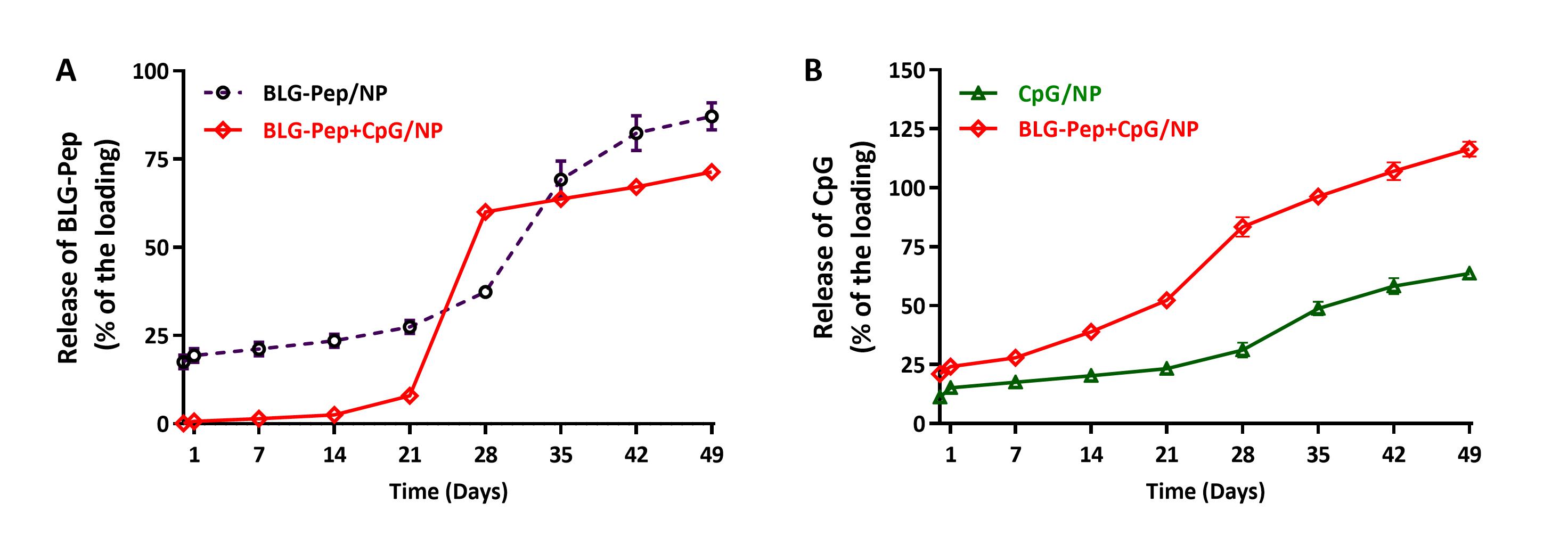

Supplement: Supplementary file 1 [file Image_1.jpeg]

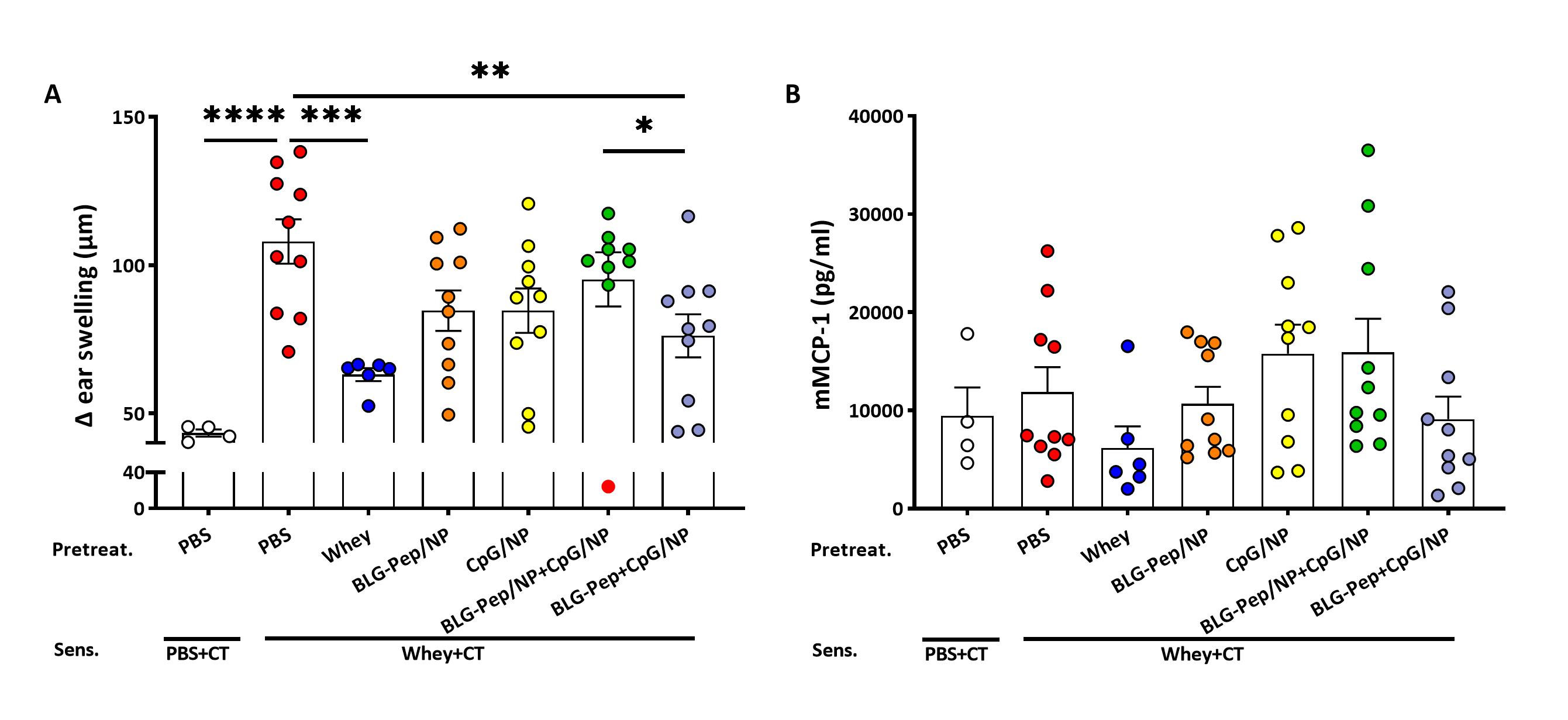

Supplement: Supplementary file 2 [file Image_2.jpeg]

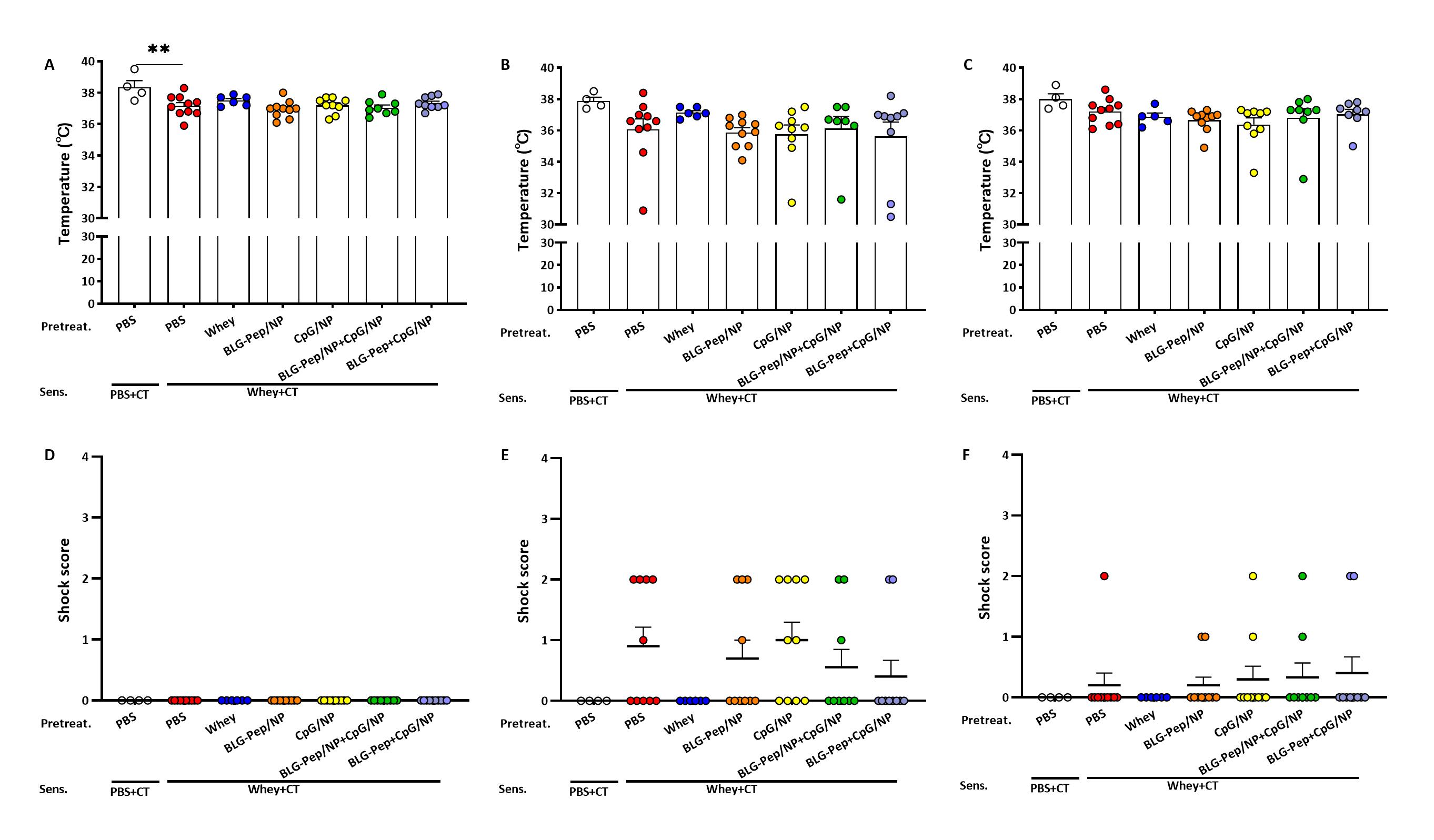

Supplement: Supplementary file 3 [file Image_3.jpeg]

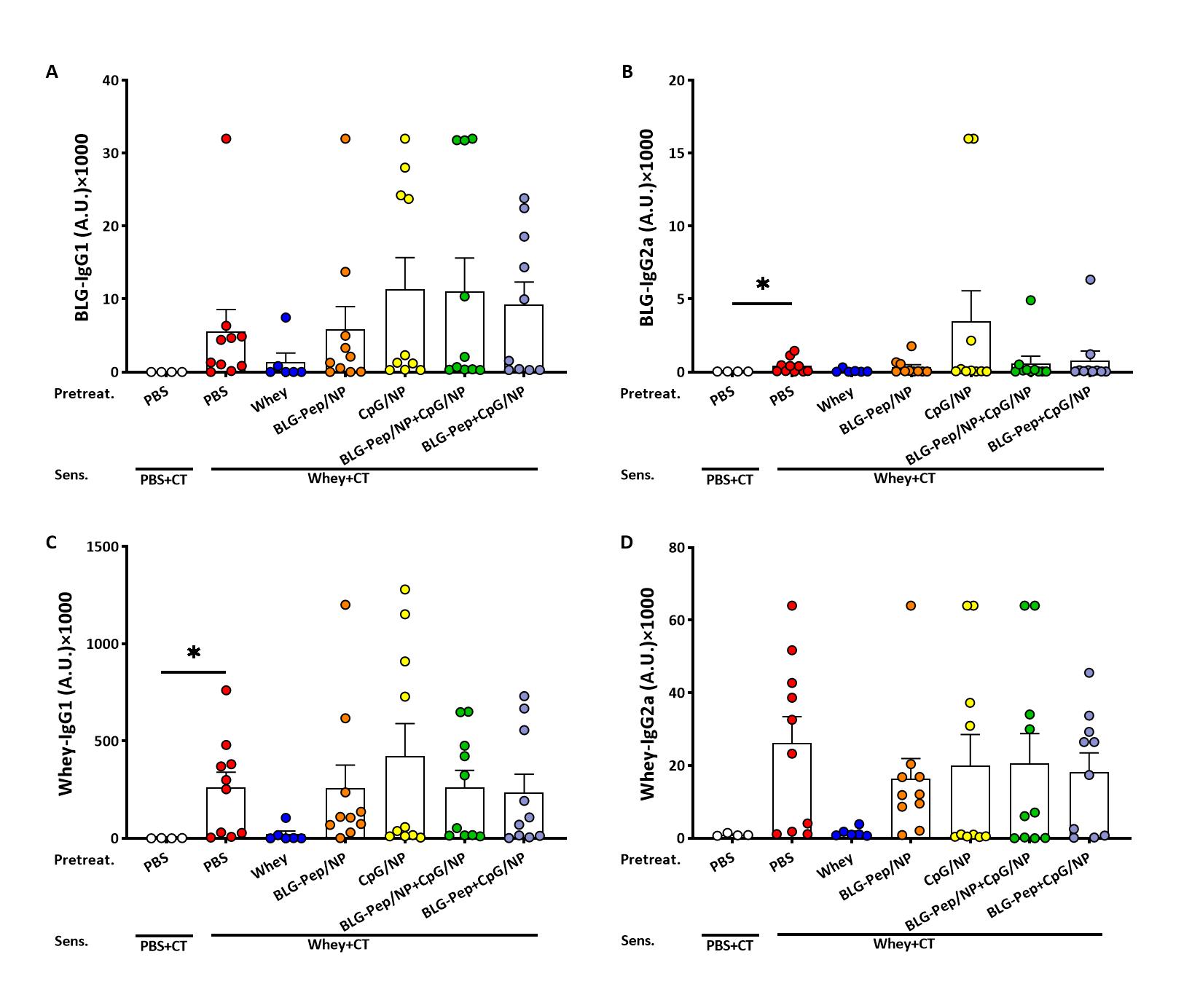

Supplement: Supplementary file 4 [file Image_4.jpeg]

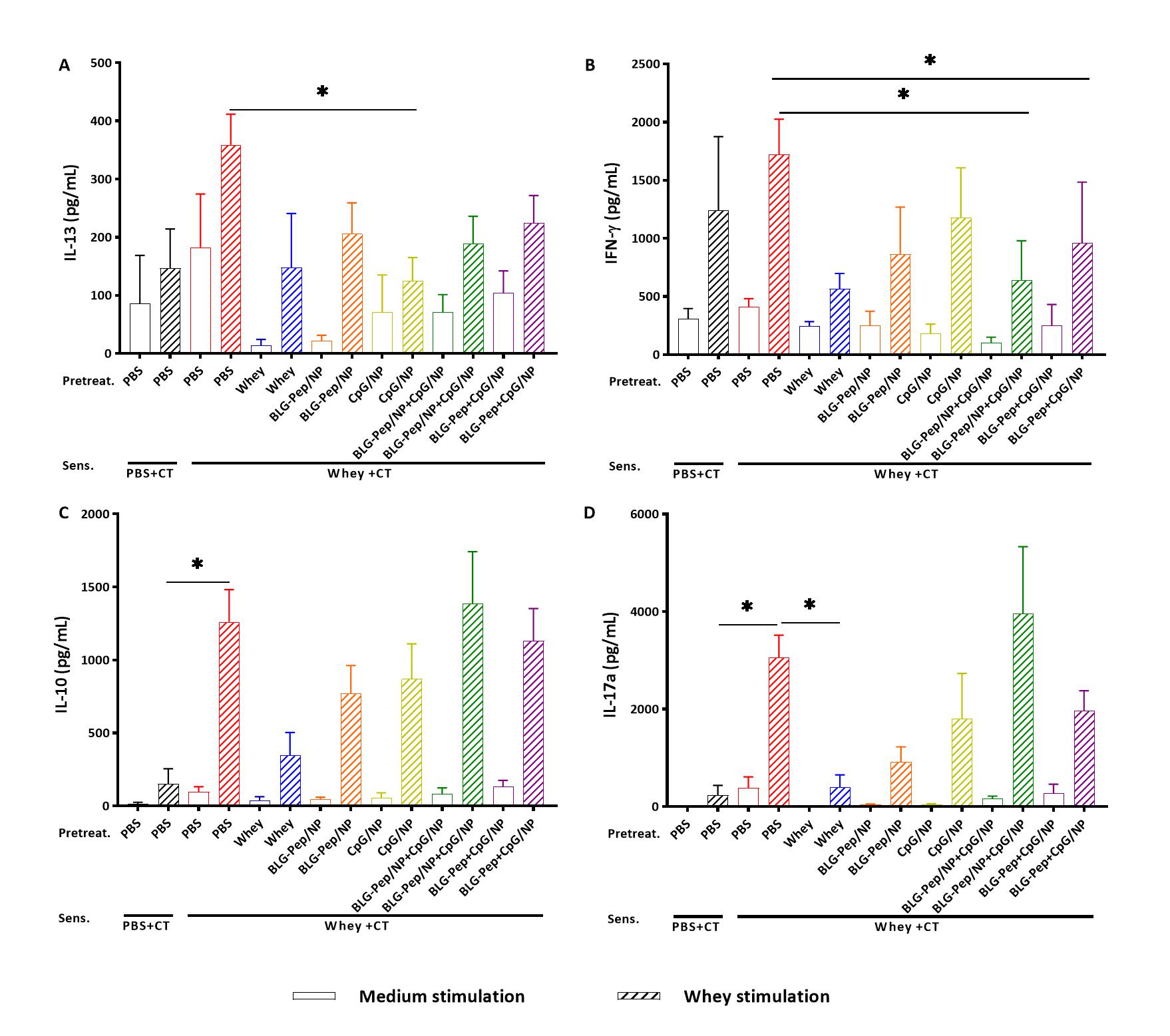

Supplement: Supplementary file 5 [file Image_5.jpeg]

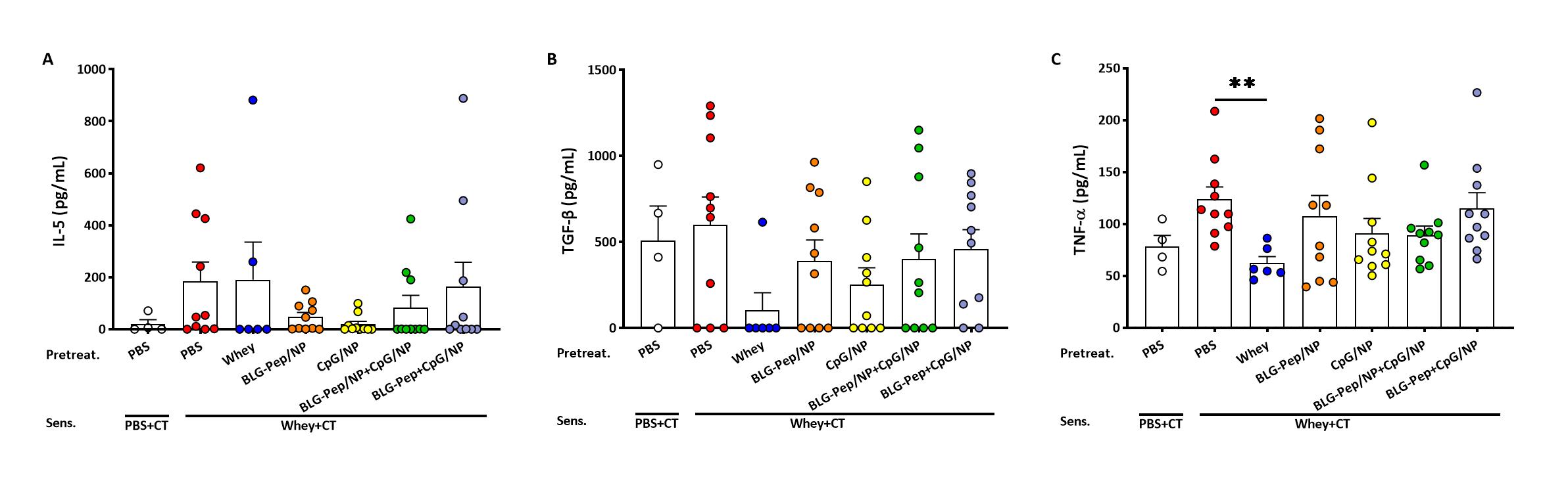

Supplement: Supplementary file 6 [file Image_6.jpeg]

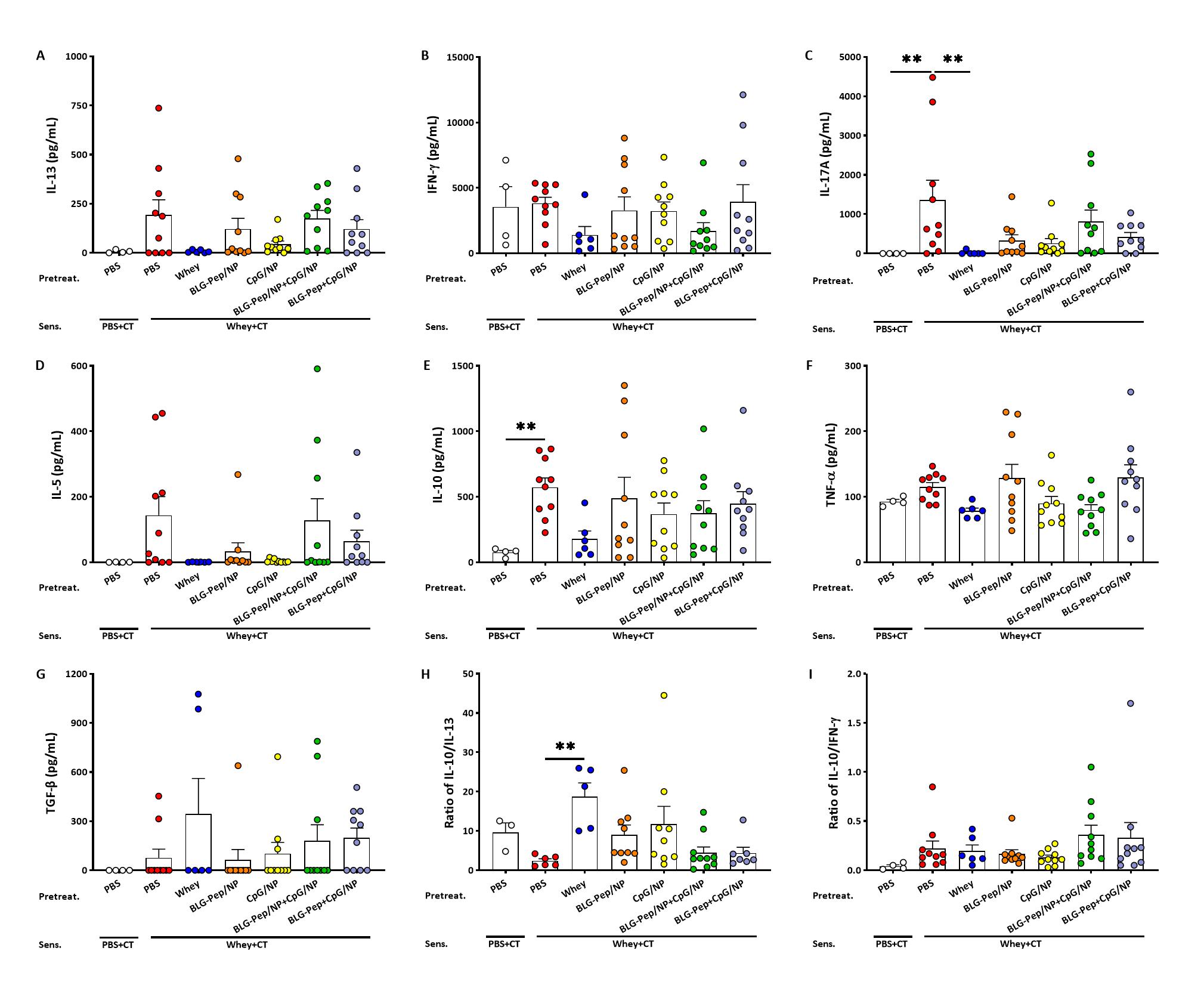

Supplement: Supplementary file 7 [file Image_7.jpeg]

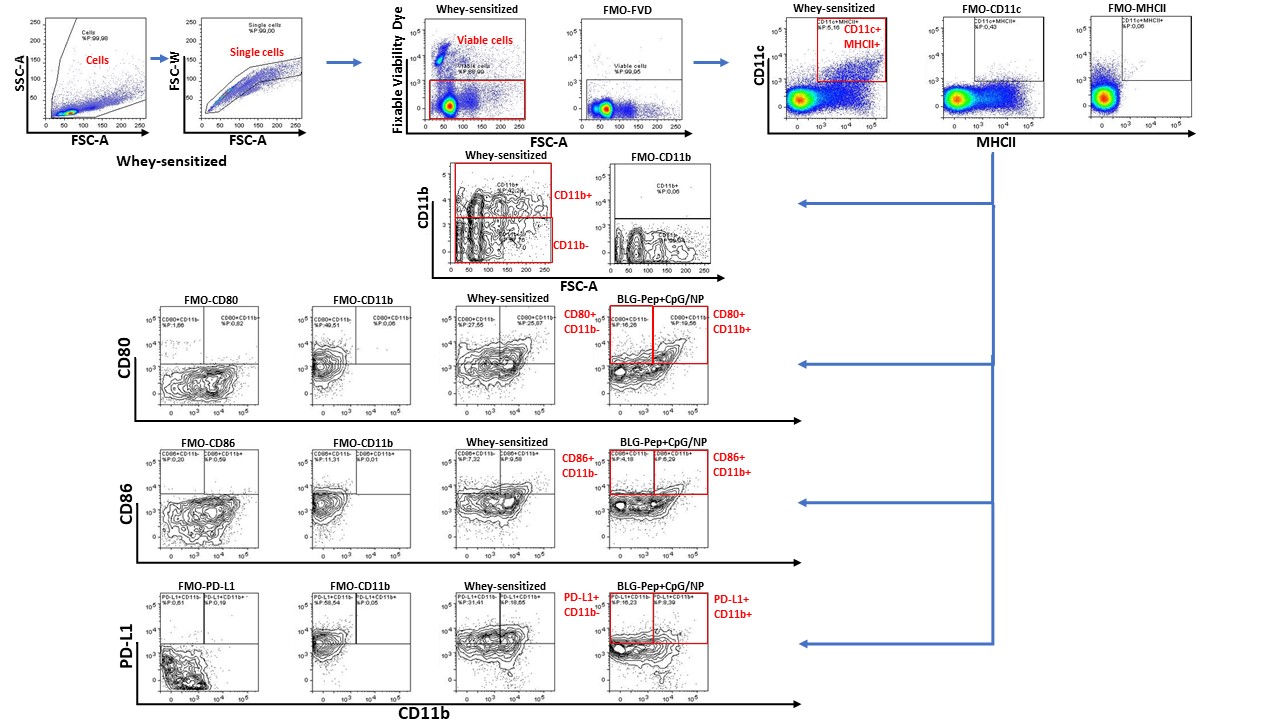

Supplement: Supplementary file 8 [file Image_8.jpeg]

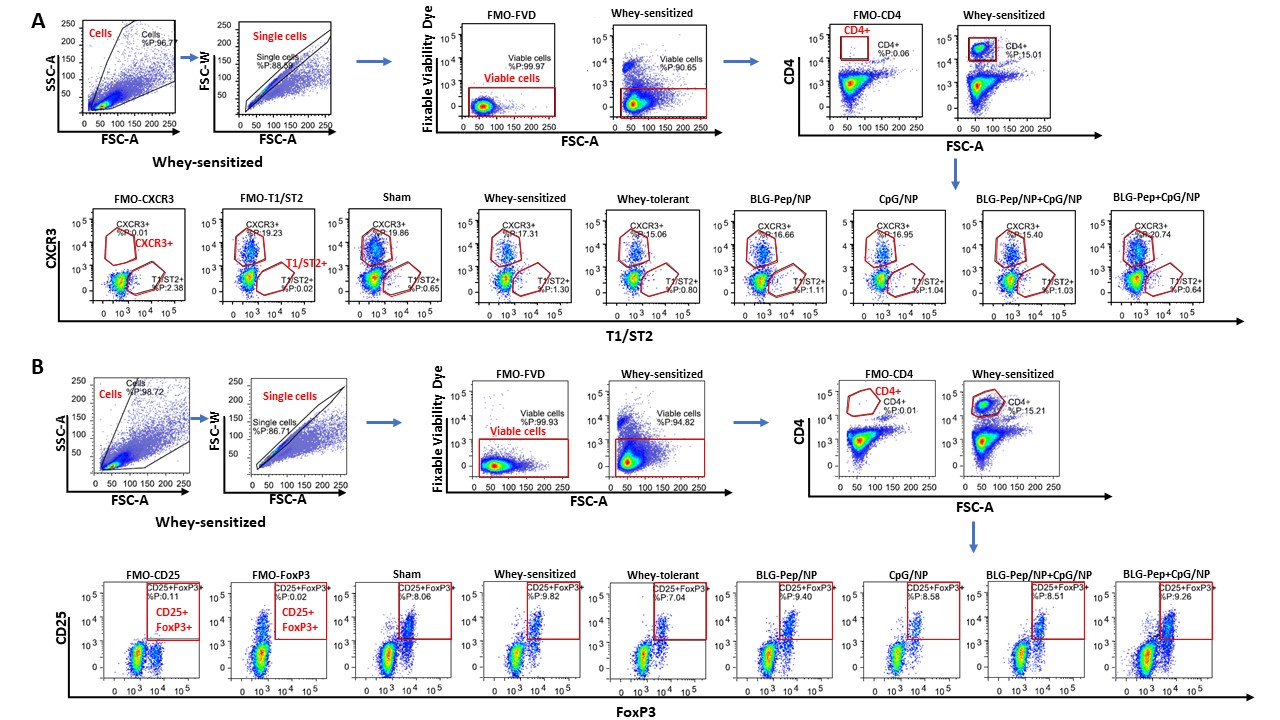

Supplement: Supplementary file 9 [file Image_9.jpeg]
