# Supplementary material for: Oral pretreatment with β-lactoglobulin derived peptide and CpG co-encapsulated in PLGA nanoparticles prior to sensitizations attenuates cow’s milk allergy development in mice
Source: Front Immunol. 2023 Jan 6;13:1053107. doi: 10.3389/fimmu.2022.1053107 (PMC9872660; doi:10.3389/fimmu.2022.1053107)
Supplement: Supplementary file 10 [file DataSheet_1.docx]

Supplementary Material

1. **Supplementary Data**
   1. **Preparation and characterization of BLG-peptide and/or CpG encapsulated PLGA nanoparticles**

Nanoparticles were prepared with PLGA (lactide/glycolide molar ratio 50:50, 0.32-0.48 dl/g; PURASORB PDLG 5004A, Corbion, the Netherlands) using a double emulsion solvent evaporation method (1). Briefly, 160 mg PLGA was weighed and dissolved in 4 mL dichloromethane (DCM, Biosolve BV, Valkenswaard, the Netherlands) overnight at room temperature. Next, 1) 3.2 mg BLG-Pep in 400 µL sterile PBS and 65 µg CpG in 50 µL PBS for BLG-Pep+CpG/NP or 2) 400 µL PBS and 200 µg CpG in 50 µL PBS for CpG/NP or 3) 400 µL PBS and 50 µL PBS for Empty NP, were added dropwise into 3 mL and 1 mL PLGA-solutions, respectively, and sonicated using a Sonifier S-450A (3 mm, Branson Ultrasonics B.V., Soest, the Netherlands) at 20% amplitude for 0.5 min on ice bath. The obtained two water-in-oil emulsions were combined and further sonicated (at 20% amplitude for 0.5 min on ice bath) to yield one single water-in-oil-emulsion. Subsequently, the obtained water-in oil-emulsion was added dropwise into 40 mL external aqueous phase, containing 3 w/v % polyvinyl alcohol (87-90% hydrolyzed, Mw 3,000-70,000 Da, Sigma-Aldrich, Zwijndrecht, the Netherlands) and 0.9 w/v % sodium chloride (NaCl, Sigma-Aldrich), and the obtained mixture was sonicated at 20% amplitude for 1 min on ice bath to yield the final water-in-oil-in-water emulsion. Finally, the formed emulsion was subjected to agitation for 3 h at room temperature to evaporate DCM and to obtain hardened PLGA NPs. The nanoparticle suspension was centrifuged at 20,000×g for 30 min at 4℃ and the obtained pellet was washed with 20 mL nuclease free water (not DEPC-Treated, Invitrogen, Life Technologies, Carlsbad, USA) twice prior to lyophilization using a freeze dryer (Buchi Lyovapor L-200, Hendrik-Ido-Ambacht, the Netherlands).

Next, 2 μL of the prepared NP suspensions was added into 998 μL milliQ water for characterization of nanoparticle size and polydispersity index (PDI) with Zetasizer Nano S (Malvern Instruments, Malvern, UK), or transferred into 998 μL 10 mM HEPEs buffer (pH7.4) for characterization of surface charge of the NP formulations with Zetasizer Nano-Z (Malvern Instruments, Malvern, UK) respectively.

BLG-Pep alone were encapsulated in PLGA nanoparticles as published previously (2, 3). Encapsulation efficiency of BLG-peptide in the BLG-Pep/NP was measured with a direct method using UPLC as published previously (2). Encapsulation efficiency of CpG was quantified with a direct method. In detail, around 10-15 mg lyophilized CpG/NP or BLG-Pep+CpG/NP were accurately weighed and hydrolyzed in 450 μL 0.2N NaOH solution per 5 mg NP overnight (for 17 h) at room temperature under agitation until the PLGA polymers were fully hydrolyzed. Next, 22.5 μL 20×Tris-EDTA (ThermoFisher) buffer per 5 mg NP was added into the hydrolysis solution to stop the hydrolysis and additional volume of 1×Tris-EDTA buffer was added to reach 10 mg/mL NP-equivalent of the hydrolysis solution prior to the quantification assay for CpG using Quant-iT™ OliGreen™ ssDNA Assay Kit (ThermoFisher) according to the manufacturer’s protocol.

- 1. ***In vitro* release of BLG-Pep and CpG from PLGA nanoparticles**

The release of BLG-Pep from the PLGA NP from the animal study was determined as follows. Around 10 mg of freeze dried BLG-Pep/NP, CpG/NP and 20 mg BLG-Pep+CpG/NP were accurately weighed in triplicate, prior to suspension in 500 µL phosphate saline buffer (PBS, pH7.4)-0.06 w/v % sodium azide (NaN_3_, Sigma-Aldrich) (release buffer) and incubation at 37°C on a nutating mixer. At different time points (at 30 min, day 1, 7, 14, 21, 28, 35, 42 and 49 time points respectively), samples were centrifuged at 20,000×g at 4°C and 400 µL supernatant was withdrawn. Next, 400 µL of fresh release buffer was added, the pelleted NPs were resuspended and the samples were further incubated at 37°C on the nutating mixer.

BLG-Peptide (BLG-Pep) and CpG were measured in the different release samples using established UPLC method as described previously (2) and Quant-iT™ OliGreen™ ssDNA Assay Kit (ThermoFisher) according to the manufacturer’s protocol, respectively. The percentage of released peptide cargo and CpG were calculated based on the encapsulated amount as described previously (2).

# Supplementary Figures and Tables

## Supplementary Figures

**Supplemental Figure 1.** *In vitro* release of β-lactoglobulin derived peptide (namely BLG-Pep) (A) and CpG (B) from PLGA NPs used for the animal study in PBS (pH7.4) at 37℃. The release samples were taken at 30 min, day 1, 7, 14, 21, 28, 35, 42 and 49 time points respectively. Data are presented as mean ± SD, n=3 per formulation.

**Supplemental Figure 2.** **Acute allergic skin response and serum murine mucosal mast cell protease-1 (mMCP-1).** Five days after last sensitization, mice were intradermally challenged in the ear pinnae with 10 µg whey and the acute allergic skin response was measured 60 min (A) afterwards. In addition, mMCP-1 (B) was quantified in the collected 18 h after last oral challenge with whey in mice from all groups of the animal study. Data are presented as mean ± SEM, n=9-10 per group except for the sham group, n=4 and whey-tolerant group, n=6. (A) is presented with Y axis formatted in two segments to properly show the relevant part for the window of effect. (A) is analyzed by one-way ANOVA, followed by Bonferroni's *post hoc* test for selected pairs; The outlier (in red) in the BLG-Pep/NP+CpG/NP group is excluded in (A) from statistics; (B) is analyzed with Kruskal-Wallis' non-parametric test, followed by Dunn’s *post hoc* test for selected pairs. **p*<0.05, ***p*<0.01, ****p*<0.001, *****p*<0.0001; BLG, β-lactoglobulin; CT, Cholera toxin.

**Supplemental Figure 3. Anaphylactic shock score and body temperature after intradermal challenge with whole whey protein.** Body temperature and anaphylactic shock score were recorded at 0 h (A) (D), 30 min (B) (E) and 60 min (C) (F) after intradermal whey challenge in mice from all groups of the animal study. Data are presented as mean ± SEM for n=8-10 per group except for the sham group, n=4 and whey-tolerant group, n=5-6. (A-C) are presented with Y axis formatted in two segments to properly show the relevant window for shock induced body temperature changes. (A) is analyzed with one-way ANOVA for selected pairs after log transformation, followed by Bonferroni's *post hoc* test; (B-E) are analyzed with the Kruskal-Wallis non-parametric test, followed by Dunn's *post hoc* test for selected pairs; ***p*<0.01; BLG, β-lactoglobulin; CT, Cholera toxin.

**Supplemental Figure 4. BLG- and whey-specific serum immunoglobulins levels.** BLG- (A-B) and whey-specific (C-D) IgG1 and IgG2a levels are measured in serum, which were collected 18 h after last oral challenge with whey in mice from all groups of the animal study. Data are presented as mean ± SEM for n=9-10 per group except for the sham group, n=4 and whey-tolerant group, n=6. (A-D) are analyzed with the Kruskal-Wallis non-parametric test, followed by Dunn's *post hoc* test for selected pairs; **p*<0.05; BLG, β-lactoglobulin; CT, Cholera toxin.

**Supplemental Figure 5. Cytokines production of splenocytes after *ex vivo* stimulation with medium or whole whey protein for 5 days.** Th2- (A), Th1- (B), Treg- (C), and Th17- (D) associated cytokines were measured in supernatants. Data are presented as mean ± SEM for n=9-10 per group except for the sham group, n=4 and whey-tolerant group, n=6. (A-D) are analyzed with the Kruskal-Wallis non-parametric test only for whey stimulation, followed by Dunn's *post hoc* test for selected pairs; **p*<0.05; CT, Cholera toxin.

**Supplemental Figure 6. Cytokines production of splenocytes after *ex vivo* stimulation with whole whey protein for 5 days.** Splenocytes were restimulated with medium or whole whey protein for 5 days, the medium stimulated cytokine release are subtracted. Th2- (A), Treg- (B), pro-inflammatory- (C) associated cytokines were measured in supernatants. Data are presented as mean ± SEM for n=9-10 per group except for the sham group, n=4 and whey-tolerant group, n=6. (A), (B), (C) are analyzed with the Kruskal-Wallis non-parametric test, followed by Dunn's *post hoc* test for selected pairs; ***p*<0.01; CT, Cholera toxin.

**Supplemental Figure 7. Cytokines production of splenocytes after *ex vivo* stimulation with β-lactoglobulin for 5 days.** Splenocytes were restimulated with medium or β-lactoglobulin for 5 days, the medium stimulated cytokine release are subtracted. Th2- (A and D), Th1- (B), Th17- (C),Treg- (E and G), proinflammatory (F) associated cytokines were measured in supernatants. Ratios of Treg-/Th2- associated (H) and Treg-/Th1-associated (I) cytokines were calculated. Data are presented as mean ± SEM for n=9-10 per group except for the sham group, n=4 and whey-tolerant group, n=6. (A-I) are analyzed with the Kruskal-Wallis non-parametric test, followed by Dunn's *post hoc* test for selected pairs; **p*<0.05, ***p*<0.01; CT, Cholera toxin.

**Supplemental Figure 8. Gating strategy of the flow cytometry analysis of DC from mesenteric lymph nodes (MLN).**

**Supplemental Figure 9.** **Gating strategy of the flow cytometry analysis of T helper 1 and 2 (Th1 and Th2) subsets (A) and regulatory T cells (Treg) (B) in splenocytes.**

# References

1. Iqbal M, Zafar N, Fessi H, Elaissari A. Double Emulsion Solvent Evaporation Techniques Used for Drug Encapsulation. *Int J Pharm* (2015) 496(2):173-90. Epub 2015/11/03. doi: 10.1016/j.ijpharm.2015.10.057.

2. Kostadinova AI, Middelburg J, Ciulla M, Garssen J, Hennink WE, Knippels LMJ, et al. Plga Nanoparticles Loaded with Beta-Lactoglobulin-Derived Peptides Modulate Mucosal Immunity and May Facilitate Cow's Milk Allergy Prevention. *Eur J Pharmacol* (2018) 818:211-20. Epub 2017/10/29. doi: 10.1016/j.ejphar.2017.10.051.

3. Liu M, Thijssen S, van Nostrum CF, Hennink WE, Garssen J, Willemsen LEM. Inhibition of Cow's Milk Allergy Development in Mice by Oral Delivery of Beta-Lactoglobulin-Derived Peptides Loaded Plga Nanoparticles Is Associated with Systemic Whey-Specific Immune Silencing. *Clin Exp Allergy* (2022) 52(1):137-48. Epub 2021/06/20. doi: 10.1111/cea.13967.
