# Supplementary material for: Oral pretreatment with β-lactoglobulin derived peptide and CpG co-encapsulated in PLGA nanoparticles prior to sensitizations attenuates cow’s milk allergy development in mice
Source: Front Immunol. 2023 Jan 6;13:1053107. doi: 10.3389/fimmu.2022.1053107 (PMC9872660; doi:10.3389/fimmu.2022.1053107)
Supplement: Supplementary file 11 [file DataSheet_2.docx]

## Supplementary Table

**Supplemental Table 1. Flow cytometry staining of T helper 1 (Th1) and T helper 2 (Th2) subsets from spleen**

| **Antibodies** | **Supplier** | **Cat. no.** | **Dilution** | **Buffer** |
| --- | --- | --- | --- | --- |
| CD4-BV510 | BioLegend | 100553 | 1:160 | 1% Bovine Serum Albumin (BSA)^[[1]](#footnote-1)^-Dulbecco's phosphate-buffered saline (PBS)^[[2]](#footnote-2)^ |
| T1/ST2-FITC | mdbioproducts | 101001F | 1:200 | 1% BSA-PBS |
| CXCR3-PE | ThermoFisher | 12-1831 | 1:100 | 1% BSA-PBS |

**Supplemental Table 2. Flow cytometry staining of regulatory T-cells (Treg) from spleen**

| **Antibodies** | **Supplier** | **Cat. no.** | **Dilution** | **Buffer** |
| --- | --- | --- | --- | --- |
| CD4-BV510 | Biolegend | 100553 | 1:160 | 1% Bovine Serum Albumin (BSA)^1^-Dulbecco's phosphate-buffered saline (PBS)^2^ |
| CD25-PerCP/Cy5.5 | ThermoFisher | 45-0251 | 1:1280 | 1% BSA-PBS |
| FoxP3-FITC | ThermoFisher | 11-5773 | 1:100 | Permeabilization buffer^[[3]](#footnote-3)^ |

**Supplemental Table 3. Flow cytometry staining of surface activation markers staining of dendritic cells from spleen and mesenteric lymph node**

| **Antibodies** | **Supplier** | **Cat. no.** | **Dilution** | **Buffer** |
| --- | --- | --- | --- | --- |
| MHCII-PE | ThermoFisher | 12-5322 | 1:640 | 1% Bovine Serum Albumin (BSA)^1^-PBS^2^ |
| CD11c-FITC | ThermoFisher | 11-0114 | 1:3200 | 1% BSA-PBS |
| CD11b-PE/Cy7 | ThermoFisher | 25-0112 | 1:2580 | 1% BSA-PBS |
| CD80-BV421 | BioLegend | 104726 | 1:160 | 1% BSA-PBS |
| CD86-BV510 | BioLegend | 105040 | 1:320 | 1% BSA-PBS |
| PD-L1-PerCP/eFluor710 | ThermoFisher | 46-5982 | 1:1000 | 1% BSA-PBS |

1. Bovine Serum Albumin (BSA) was purchased from Sigma-Aldrich. [↑](#footnote-ref-1)
2. Dulbecco's phosphate-buffered saline (PBS) was purchased from Sigma-Aldrich. [↑](#footnote-ref-2)
3. Permeabilization buffer was prepared using the FoxP3/Transcription Factor Staining Buffer Set (ThermoFisher) according to the protocol provided by the supplier. [↑](#footnote-ref-3)
